# Reliability and Mental Health Correlates of a Single-Item Measure of Self-Rated Mental Health (SRMH) in the Chinese Context

**DOI:** 10.3390/healthcare12010122

**Published:** 2024-01-04

**Authors:** Hong Wang Fung, Stanley Kam Ki Lam, Wai Tong Chien, Henry Wai-Hang Ling, Zi Yi Wu, Colin A. Ross, Anson Kai Chun Chau

**Affiliations:** 1 Department of Social Work, Hong Kong Baptist University, Kowloon Tong, Hong Kong SAR, China; 2 The Nethersole School of Nursing, Faculty of Medicine, The Chinese University of Hong Kong, Hong Kong SAR, China; stanleylam@cuhk.edu.hk (S.K.K.L.); wtchien@cuhk.edu.hk (W.T.C.); 3 The Department of Social Work and Social Administration, The University of Hong Kong, Hong Kong SAR, China; henrylinglwh@gmail.com; 4 Yuli Hospital Ministry of Health and Welfare, Hualien 981, Taiwan; u88877@gmail.com; 5 The Colin A. Ross Institute for Psychological Trauma, Richardson, TX 75080, USA; rossinst@rossinst.com; 6 Department of Psychology, The Chinese University of Hong Kong, Hong Kong SAR, China; ansonckc123@link.cuhk.edu.hk; 7 Institute of Health Equity, The Chinese University of Hong Kong, Hong Kong SAR, China

**Keywords:** assessment, epidemiology, mental health, public health, reliability, self-rated mental health (SRMH)

## Abstract

The use of single-item measures of self-rated mental health (SRMH) has been increasingly valued in epidemiologic research. However, little is known about the reliability and mental health correlates of SRMH in Chinese populations. This study examined the reliability and mental health correlates of SRMH in three Chinese samples. We analyzed data collected from two convenience samples of Chinese adults from Hong Kong and/or Taiwan (Sample 1: N = 205; Sample 2: N = 377), and a random sample of Taiwan psychiatric inpatients (Sample 3: N = 100). Our results showed that the single-item measure of SRMH had moderate to good test–retest reliability (intraclass correlation [ICC] = 0.75) in Sample 1 and acceptable reliability between the self-report and interviewer-administered versions (ICC = 0.58) in Sample 3. It had a high positive correlation with self-esteem and a moderately high negative correlation with depression. It also had a consistently negative correlation with borderline personality disorder symptoms and post-traumatic stress disorder symptoms. The SRMH score was also associated with psychiatric service usage. These findings contribute to the body of knowledge regarding the use of a single-item measure of SRMH to assess overall self-perceived mental health in Chinese communities.

## 1. Introduction

Mental health problems are significant public health issues not only associated with individual suffering but also significant family and societal consequences. For either public health research or general screening in the community, self-report measures are probably the most commonly used tools to assess mental health or mental disorders. For these purposes, scales with fewer items are useful and are increasingly valued, whereas scales with many items can provide comprehensive information but have disadvantages in terms of practicality and acceptability for research and clinical screening. In particular, Smits et al. [1] and Haroz et al. [2] have emphasized the importance of developing reliable short mental health measures, and have described the disadvantages of using lengthy scales, such as an increasing response burden of the responder, and being difficult to complete for people with shorter attention spans. In addition, lengthy scales may affect the quality of the data obtained because some participants might not be willing to participate or to complete all of the scale items. 

Single-item measures of self-rated health (SRH) or mental health (SRMH) have received increasing attention in recent epidemiologic studies [3,4,5]. The literature review of Ahmad et al. [6] suggested that single-item measures of SRMH were first used in the 1970s, and subsequently have been included as part of the sets of questions in measures which have been used in different national epidemiologic studies. Different versions of the single-item measures of SRMH often have similar meanings, for example, “In general, would you say your mental health is…” and “How do you rate your mental health at the present time?”. Ahmad et al. [6] conducted a review to examine how single-item measures of SRMH were correlated with other mental health measures in public health research. Among 57 included studies, they reported that “SRMH correlated moderately with the following mental health scales: Kessler Psychological Distress Scale, Patient Health Questionnaire, mental health subscales of the Short-Form Health Status Survey, Behaviour and Symptom Identification Scale, and World Mental Health Clinical Diagnostic Interview Schedule” (p. 1). For example, in one of the studies, the single-item SRMH was correlated significantly with three different measures of depressive symptoms (r = 0.42 to 0.50) [7]. Studies examining SMRH across ethnic groups have shown that there are cultural and ethnic differences in the understanding and reporting of SRMH. For instance, mental health problems other than major depression have shown a heterogeneous relationship with SRMH in different ethnic groups [8,9]. Therefore, the findings in Ahmad et al. [6]’s review might not be generalizable to Chinese populations.

Although there is increasing evidence about their usefulness, two major research gaps concerning the use of single-item measures of SRMH require attention and further investigation. First, these single-item measures are commonly used in population surveys, but little is known about their reliability, including the test–retest reliability and reliability between the self-report and interviewer-administered versions. We are not aware of any study reporting the reliability of single-item measures of SRMH. Second, there is insufficient evidence or understanding about the mental health correlates of SRMH, in Chinese and other Asian contexts. Recently, single-item measures of SRMH have been used in several Chinese studies, e.g., [10,11]. However, few studies have evaluated their reliability or correlations with other well-validated multi-item mental health measures in Chinese societies. Only one study has reported a significant negative correlation (r = −0.48, *p* < 0.001) between a single-item measure of SRMH (i.e., “How would you rate your overall mental or emotional health?” and depressive symptoms in a sample of older Chinese Americans (N = 108) [12]. To address these two research gaps, this study examined the reliability of a single-item measure of SRMH (i.e., test–retest reliability and reliability between the self-report and interviewer-administered versions) and how it would correlate with measures of other mental health variables (including common psychiatric symptoms) among Chinese research participants. We hypothesized that the single-item measure of SRMH used would have acceptable reliability (i.e., intraclass correlation coefficient > 0.5) and significant correlations with measures of common mental health symptoms, including depression and anxiety. We also hypothesized that the single-item measure of SRMH would be correlated with psychiatric service usage. In summary, the primary objective of this study is to establish the reliability and validity of the Chinese version of the SRMH.

## 2. Materials and Methods

### 2.1. Participants and Settings

This study analyzed data collected from three samples of Chinese participants mainly from Hong Kong and Taiwan. 

*Sample 1: An online convenience sample of young adults mainly from Hong Kong and Taiwan.* Participants were recruited in a project conducted by the Achievement Foundation, a registered charitable institution in Hong Kong; and approval was obtained from the executive committee of this organization. During the period between April 2021 and July 2021, this project aiming to investigate mental health problems in a convenience sample of Chinese young adults aged 18–24; participants (N = 205) were recruited through online social media platforms to participate in a web-based survey on trauma and related mental health problems. Participants: (1) were able to read and write Chinese; (2) had access to the internet; and (3) were willing to participate after providing informed consent on the first instruction page and then completed the anonymous online survey. There were no exclusion criteria. Email addresses of participants were obtained in order to conduct a retest at a one-week interval. The methodology, sampling and results of the study have been reported elsewhere [13]. In Sample 1, N = 205 young adults participated in the web-based survey. Most participants came from Taiwan (65.4%) and Hong Kong (29.3%), while 5.4% reported their place of residence as “other”. Most of them were female (84.9%).

*Sample 2: A convenience sample of Hong Kong adults who received primary care (traditional Chinese medicine [TCM]) services in the past three months.* Participants were recruited from a project which was approved by the institutional review board of the Chinese University of Hong Kong. This project examined psychosocial and mental health experiences in a convenience sample of Hong Kong adults (N = 377). In 2022, the project recruited participants through Hong Kong-based channels (e.g., social media platforms and local TCM clinics) to participate in a web-based survey that included measures of various mental health problems. Participants had to (1) be aged 18 or above, (2) be Hong Kong Chinese residents who received TCM services in the past three months, and (3) be willing to participate after providing informed consent on the first instruction page and then complete the anonymous online survey. Only participants who self-reported that they had been diagnosed with a learning or reading disorder, dementia, and/or cognitive impairments were excluded. The methodology, sampling and results of the study have been reported elsewhere [14]. In Sample 2, N = 377 Hong Kong community health (TCM) service users participated in the web-based survey. As in the Taiwan sample, most of them were female (80.9%).

*Sample 3: A random sample of Taiwan psychiatric inpatients diagnosed with schizophrenia spectrum disorders.* In 2021, 100 inpatients with schizophrenia spectrum disorders were recruited from a total of 486 inpatients staying one to several years in the largest psychiatric hospital in Taiwan, the Yuli Hospital of the Ministry of Health and Welfare. Ethics approval was obtained from the Institutional Review Board of this hospital. Participants were aged 18 or above, agreed to provide written consent and to participate, and had a clinical diagnosis of schizophrenia spectrum and other psychotic disorders according to DSM-5 criteria. Patients were excluded if they had (1) difficulties in communication because of cognitive impairments; (2) a clinical diagnosis of dementia; (3) speech or hearing impairments; or (4) been discharged from the hospital before the assessments could be completed. In this project, an interviewer (an occupational therapist) used an online randomizer to select potential participants and then invited them to participate in the survey and a follow-up structured interview. Once informed written consent had been obtained, participants were invited to complete a questionnaire consisting of several self-report measures of trauma and mental health symptoms. Ten days after completion of the self-report measures, the interviewer conducted a structured interview with each participant using selected sections of the Dissociative Disorders Interview Schedule (DDIS) and the Psychotic Symptom Rating Scales (PSYRATS). In sample 3, 100 Taiwan psychiatric inpatients with a clinical diagnosis of schizophrenia (N = 79) or schizoaffective disorder (N = 21) participated in the survey and structured interview. More than half of them were male (55%). 

The sociodemographic and clinical characteristics of each of the three samples are summarized in Table 1. 

### 2.2. Measures

This study examined the reliability, including the test–retest reliability and reliability between the self-report and interviewer-administered versions, of SRMH and its correlation with measures of other mental health symptoms in the above-mentioned three Chinese samples; the single-item measure of SRMH was administered to all three Chinese samples. In addition to the SRMH, several mental health measures of self-esteem, depression, anxiety, post-traumatic stress disorder (PTSD) symptoms, and borderline personality disorder (BPD) symptoms, and psychotic symptoms were used and are described as follows:

*Single-item measure of self-rated mental health (SRMH) (possible range: 1 to 5).* The single-item measure of SMRH asked, “How would you rate your overall mental health?” (1 = poor, 2 = fair, 3 = good, 4 = very good, 5 = excellent) [6]. As mentioned, this single-item measure and its modified versions have been used in previous studies, including in Chinese populations [12], and found to be correlated moderately with other well-established measures of depression and psychological distress [6]. The single-item measure of SMRH has also been used in some recent studies [4]. However, the Chinese version has not been psychometrically evaluated; higher ratings of the single item would indicate a better self-rated mental health. Participants in all three samples completed this single-item measure.

*Single-item measure of self-esteem (SISE) (possible range: 1 to 9).* The SISE, which asked, “How satisfied are you with yourself?” (1 = very dissatisfied, 9 = very satisfied), is a valid single-item self-report measure of self-esteem [15]. The literature indicates that both multi-item and single-item measures of self-esteem have very consistent relationships with other psychological and health variables [16]. In Sample 1 of this study, in which N = 116 participants completed a retest after an average of 9.32 days (SD = 3.97), the Chinese version of the SISE was found to have good test–retest reliability (ICC = 0.82, *p* < 0.001), and good construct validity (a moderate negative correlation with depression). Participants in all samples completed the SISE. 

*Patient Health Questionnaire-9 (PHQ-9) (possible range: 0 to 27).* The PHQ-9 is a self-report measure of DSM depressive symptoms. The PHQ-9 was reported to have good internal consistency (α = 0.86), test–retest reliability (r = 0.84) and concurrent validity with the Beck Depression Inventory [17,18,19]. The Chinese version of the PHQ-9 also demonstrated excellent internal consistency (α = 0.91) and good diagnostic validity (the sensitivity was 81% and the specificity was 98% when a cutoff of 15 was used to detect major depressive disorder) [20]. The Chinese version of the PHQ-9 has also been found to be a reliable and valid scale across gender and age groups in a recent study [21]. Participants in Sample 1 (α = 0.92; M = 8.01; SD = 6.39; Skewness = 1.04; Kurtosis = 0.57) and Sample 2 (α = 0.92; M = 11.19; SD = 6.98; Skewness = 0.30; Kurtosis = −0.84) completed the Chinese version of the PHQ-9. A sample item is “Feeling down, depressed, or hopeless”.

*The PTSD subscale of the International Trauma Questionnaire (ITQ) (possible range: 0 to 24).* The ITQ is originally a 18-item self-report measure of ICD-11 complex PTSD symptoms; a 6-item subscale can be particularly used to assess classical PTSD symptoms [22]. The Chinese version of the ITQ also has good internal consistency and validity [23]. Participants in Sample 2 completed the Chinese version of the ITQ (α = 0.89; M = 9.23; SD = 6.29; Skewness = 0.13; Kurtosis = −0.98). A sample item is “Being “super-alert”, watchful, or on guard”?

*PTSD Checklist for DSM-5 (PCL-5) (possible range: 0 to 80).* The PCL-5 is a 20-item self-report measure of DSM-5 PTSD symptoms; it has excellent internal consistency (α = 0.94), good test–retest reliability (r = 0.82), good convergent validity (r = 0.74 to 0.85) and had excellent diagnostic validity when a cutoff of 31 was used (sensitivity = 94.1%, specificity = 93.9%) [24,25]. The Chinese version of the PCL-5 also had excellent internal consistency (α = 0.95) and acceptable diagnostic validity when a cutoff of 49 was used (sensitivity = 70.6%, specificity = 72.7%) [26]. Participants in Sample 1 (α = 0.96; M = 34.19; SD = 20.26; Skewness = 0.06; Kurtosis = −0.95) and Sample 3 (α = 0.95; M = 40.32; SD = 13.49; Skewness = −0.61; Kurtosis = −0.53) completed the Chinese version of the PCL-5. A sample item is “Repeated, disturbing, and unwanted memories of the stressful experience”?

*Borderline Personality Disorder Section of the Self-Report Dissociative Disorders Interview Schedule (DDIS-BPD) (possible range: 0 to 9).* The DDIS-BPD is a 9-item section from the DDIS, which is an interviewer-administered structured diagnostic interview [27]. The DDIS-BPD, which includes nine yes/no/unsure items, is designed to assess the nine BPD symptoms according to DSM-5 rules, and has been used in many studies [28,29,30,31]. The Chinese version of the self-report DDIS-BPD (SR-DDIS-BPD) was found to have good convergent validity with the 20-item Taiwan version of the Borderline Personality Inventory, good construct validity with other mental health symptoms, and acceptable diagnostic validity when a cutoff of 5 was used (sensitivity = 95.2%, specificity = 64.9%); it was also demonstrated to have good concurrent validity as it could discriminate between patients with and without a BPD diagnosis [32]. Participants in all Sample 1 (M = 2.31; SD = 2.32; Skewness = 0.76; Kurtosis = −0.42) and Sample 2 (M = 1.87; SD = 2.32; Skewness = 1.21; Kurtosis = 0.55) completed the DDIS-BPD as a self-report measure and those in Sample 3 completed the DDIS-BPD in the structured interview (M = 1.32; SD = 1.36; Skewness = 1.26; Kurtosis = 1.83). A sample item is “Frantic efforts to avoid real or imagined abandonment”.

*The Dissociative Features Subsection of the DDIS (DDIS-DF).* The DDIS-DF is a 16-item subsection that is particularly designed to assess psychoform dissociative symptoms, and this measure can identify dissociative pathology very well [27,28]. The Chinese version of this measure also has good validity [30]. To assess dissociative symptoms, the DDIS-DF was administered as a self-report measure in Sample 2 (M = 1.07; SD = 1.57; Skewness = 2.21; Kurtosis = 5.80) and as part of a structured interview in Sample 3 (M = 6.99; SD = 3.53; Skewness = 0.48; Kurtosis = −0.44). A sample item is “Do you ever feel that there is another person or persons inside you”?

*Psychotic Symptom Rating Scales (PSYRATS).* The PSYRATS is a 17-item semi-structured interview for auditory hallucinations and delusions [33]. The Chinese version of the PSYRATS had excellent interrater reliability (ICC = 0.92 to 0.95), good test–retest reliability (ICC = 0.81 to 0.82), very satisfactory content validity, and good convergent validity with the Positive and Negative Syndrome Scale [34]. Participants in Sample 3 was interviewed using Chinese version of the PSYRATS (Positive Syndrome subscale: α = 0.88; M = 23.57; SD = 6.06; Skewness = −1.04; Kurtosis = 1.23; Negative Syndrome subscale: α = 0.89; M = 10.05; SD = 4.03; Skewness = −0.83; Kurtosis = 0.48).

### 2.3. Data Analysis

The data were analyzed using SPSS 22.0. This study examined the reliability of the single-item measure of SRMH by calculating its test–retest reliability in Sample 1 and interrater reliability in Sample 3 using intraclass correlation coefficients (ICC) (two-way mixed effects, absolute agreement) according to Koo and Li [35]’s guideline; ICC values below 0.05, between 0.5 and 0.75, between 0.75 and 0.9, and greater than 0.90 were considered to be poor, moderate, good, and excellent reliabilities. In this study, an ICC value above 0.5 was considered to be acceptable. In addition, we examined the mental health correlates of the single-item measure of SRMH using Spearman’s correlation coefficients. Finally, we examined whether SRMH would be associated with psychiatric service utilization using Chi-square tests—in Sample 1, participants were asked if they “had seen a psychiatrist” in the past 12 months; in Sample 2, participants were asked if they were currently seeing a psychiatrist.

## 3. Results

### 3.1. Reliability 

In Sample 1, a subgroup of participants (N = 116) completed the retest after an average of 9.32 days (SD = 3.97). The single-item measure of SRMH had moderate to good test–retest reliability (ICC = 0.75, 95% CI: 0.65–0.82, *p* < 0.001). 

In Sample 3, the single-item measure of single-item SRMH had moderate reliability between the self-report version and the interviewer-administered version (ICC = 0.58, 95% CI: 0.43–0.70, *p* < 0.001). Notably, both versions of the single-item SRMH utilized identical wording; the only difference was the mode of completion, with one being a paper questionnaire and the other being verbally asked by the interviewer. Therefore, the results here indicated a moderate test–retest reliability comparing the self-report version and the interviewer-administered version of the single-item SRMH.

### 3.2. Demographic and Mental Health Correlates

The relationships of the single-item measure of SMRH with age, gender, and other mental health variables are indicated in Table 2. SRMH was not correlated with age or gender across the samples. 

The findings indicated that the single-item measure of SMRH had a strong to very strong positive correlation with self-esteem (Samples 1–3: rho = 0.69 to 0.82, *p* < 0.001), while it had a strong negative correlation with depression (Sample 1: rho = −0.66, *p* < 0.001, Sample 2: rho = −0.59, *p* < 0.001). It also had a weak to moderate negative correlation with PTSD symptoms (Samples 1–3: rho = −0.32 to −0.52, *p* < 0.001) and BPD symptoms (Samples 1–3: rho = −0.22 to −0.48, *p* < 0.05). Its correlation with dissociative symptoms was significant in Sample 2 (rho = −0.25, *p* < 0.001) but not in the inpatient sample (rho = −0.10, *p* = 0.345). The single-item measure of SMRH did not have a significant correlation with hallucinations (rho = −0.03, *p* = 0.742) or delusions (rho = −0.18, *p* = 0.068) in the inpatient sample. 

In Sample 1, 45 participants (22.0%) reported that they “had seen a psychiatrist” in the past year. A chi-square test indicated that they were more likely to rate their own mental health as “poor” or “fair” on the SMRH than those who did not see a psychiatrist in the past year (60.0% vs. 25.6%), χ2 (1) = 18.72, *p* < 0.001).

In Sample 2, 42 participants (11.1%) reported that they were currently seeing a psychiatrist. A chi-square test indicated that they were more likely to rate their own mental health as “poor” or “fair” on the SMRH than those who were not currently seeing a psychiatrist (69.0% vs. 40.3%), χ2 (1) = 12.55, *p* < 0.001).

## 4. Discussion

This study aimed to examine the reliability and construct validity by investigating the reliability and mental health correlates of the Chinese version of the single-item measure of SRMH. The findings demonstrated that the single-item measure of SRMH had moderate to good test–retest reliability and acceptable agreement between the self-report version and the interviewer-administered version in our Chinese samples. In addition, the single-item measure of SRMH also had a strong to very strong positive correlation with self-esteem in all three Samples, a strong negative correlation with depression (in Sample 1 and Sample 2), and a weak to moderate negative correlation with PTSD and BPD symptoms (Sample 1, Sample 2 and Sample 3). The single-item measure of SRMH was also associated with self-report psychiatric service utilization in Sample 1 and Sample 2. These results indicate that the single-item measure of SRMH can be reliably used in Chinese populations to assess overall mental health.

This study contributes to the increasing body of knowledge on the use of a single-item measure of SRMH; previous studies did not establish its reliability and/or did not examine its mental health correlates, especially in Chinese populations, except for one study in older Chinese Americans in 2016 [12]. The literature revealed that cultural and ethnic differences might be observed when measuring SRMH [8,9], and previous findings on the SRMH may not be generalizable to Chinese populations. Therefore, the present study contributes to the literature by showing that the single-item measure of SRMH can be reliably used and is correlated with measures of other mental health variables in Chinese populations. 

The single-item measure of SRMH was found to be reliable and positively correlated with self-esteem, and negatively correlated with common mental health symptoms (e.g., depression and PTSD symptoms) and with self-report psychiatric service utilization in the Chinese samples. These findings suggest that the single-item measure of SRMH can be used as a public health measure to assess self-perceived general mental health among Chinese people, and can reflect ones’ overall mental well-being and correlate with other mental health conditions such as depression and anxiety, as indicated in this study.

Since early identification of mental health problems is important, regular mental health screening is recommended in health and social service settings. As the single-item measure of SRMH is very short and easily administered and has been found to be strongly associated with common measures of mental health conditions, this single-item measure can be useful and acceptable in a general health screening survey and in clinical and community service settings to facilitate early recognition of population-wide mental well-being. The SRMH measure could assist in early identification of mental healthcare needs. Whenever a person self-perceives his/her overall mental health or well-being as “poor” or “fair”, a detailed and comprehensive follow-up mental health assessment can be recommended. 

It should be noted that the single-item measure of SRMH in psychiatric inpatient settings (Sample 3) might not perform as well as in community care settings (Sample 1 and Sample 2). The SMRH was not associated with dissociation, hallucinations, or delusions in Sample 3. One possible reason for this is that inpatients with schizophrenia or schizoaffective disorder might have relatively poor insight into rating their own mental health. 

This study has several limitations. First, we relied on self-report data in Sample 1 and Sample 2 and this method is usually considered to have certain disadvantages (e.g., people providing invalid responses, social desirability bias, response bias) [36,37]. Second, Sample 1 and Sample 2 were convenience samples and were not representative of the general population, and therefore future studies should further evaluate the use of single-item measure of SMRH in different Chinese populations. Third, we analyzed cross-sectional data only, and we could not examine the predictive ability of SMRH for detecting subsequent mental health problems; future longitudinal studies will be required to examine whether the single-item measure of SRMH can predict mental health problems and/or service utilization. Fourth, while we examined the mental health correlates of the SRMH, some other indicators of mental well-being, like life satisfaction and meaning in life, were not considered in our samples. Finally, this study drew upon a selection of the literature that includes studies published more than five years ago. Notably, certain measures utilized in this research were psychometrically evaluated over five years ago. Therefore, future investigations into the clinical and public health utility of the single-item measure of SMRH should incorporate more recent studies to ensure the most up-to-date understanding and applicability of the measure.

## 5. Conclusions

This study demonstrated that the single-item measure of SRMH is a reliable measure and is significantly correlated with common valid measures of mental health problems in Chinese people. This easily implemented assessment tool can be included in public health studies and general health screening or assessments to investigate overall mental well-being in Chinese populations. Further studies are required to examine whether measures of SRMH can predict mental health outcomes and the need for psychiatric care and services.

## Figures and Tables

**Table 1:** Sample characteristics of Sample 1, Sample 2 and Sample 3.

	Sample 1 (N = 205)	Sample 2 (N = 377)	Sample 3 (N = 100)
**Descriptions**	A convenience sample of young Chinese-speaking adults aged between 18 to 24	A convenience sample of Hong Kong adults who received traditional Chinese medicine services (TCM) in the community	A random sample of psychiatric inpatients with schizophrenia or schizoaffective disorder
**Current locations**			
Hong Kong	29.3%	100%	0%
Taiwan	65.4%	0%	100%
Others	5.4%	0%	0%
**Age—Mean (SD)**	21.2 (1.92)	40.49 (12.58)	58.3 (7.31)
**Age—Range**	18 to 24	18 to 64	36 to 71
**Gender—Female %**	84.9%	80.9%	45%
**Education level**			
High school or below	16.1%	31.3%	N/A
Associate degree or equivalent	15.1%	15.6%	N/A
Bachelor’s degree	64.9%	35.0%	N/A
Postgraduate degree or above	3.9%	18.0%	N/A
**Employment/student status**			
Full-time employed	18.0%	63.7%	N/A
Part-time employed	28.8%	13.8%	N/A
Self-employed	N/A	N/A	N/A
Unemployed and homemakers	53.2%	22.5%	N/A
Students	73.2% #	N/A	N/A
**Service usage**	A total of 23.4% reported that they were currently seeking professional help for emotional or mental health problems; 22.0% had seen a psychiatrist in the past year	All participants received TCM services (primary care) in the past three months; 11.1% reported themselves as currently seeing a psychiatrist.	All of them were long-term institutionalized inpatients
**Single-item measure of self-rated mental health (test/retest @)**			
Poor	7.3%/7.8%	7.7%	17.0%/19.0%
Fair	25.9%/19.0%	35.8%	15.0%/21.0%
Good	30.7%/33.6%	26.0%	47.0%/50.0%
Very Good	32.7%/36.2%	24.1%	13.0%/8.0%
Excellent	3.4%/3.4%	6.4%	8.0%/2.0%

Notes: # In Sample 1, employment and student status were asked differently using two separate questions, and therefore a given participant may be student and being employed at the same time (i.e., the sum of all percentages is greater than 100%) @ In Sample 1, only N = 116 participants completed the retest.

**Table 2:** Correlations between the single-item measure of self-rated mental health (SRMH) and other mental health variables in three samples.

	Sample 1 (N = 205)	Sample 2 (N = 377)	Sample 3 (N = 100)
Variables	SRMH	SRMH	SRMH
Age	0.09	0.67 ***	−0.09
Gender (Female)	−0.03	−0.09	0.09
Self-esteem	0.75 ***	0.69 ***	0.82 **
Depressive symptoms	−0.66 ***	−0.60 ***	-
Post-traumatic stress disorder symptoms	−0.52 ***	−0.38 ***	−0.32 **
Borderline personality disorder symptoms	−0.48 ***	−0.46 ***	−0.22 *
Psychoform dissociative symptoms	-	−0.25 ***	−0.10
Hallucinations	-	-	−0.03
Delusions	-	-	−0.18

* *p* < 0.050 ** *p* < 0.010 *** *p* < 0.001. Note: Correlations with gender are point-biserial correlations. The remaining correlations are Spearman’s correlations.

## Data Availability

Data will be made available upon request to the corresponding author.

## References

[1] Smits N., Cuijpers P., Beekman A.T., Smit J.H. (2007). Reducing the length of mental health instruments through structurally incomplete designs. Int. J. Methods Psychiatr. Res..

[2] Haroz E.E., Kane J.C., Nguyen A.J., Bass J.K., Murray L.K., Bolton P. (2020). When less is more: Reducing redundancy in mental health and psychosocial instruments using Item Response Theory. Glob. Ment. Health.

[3] Stubbs J.M., Achat H.M. (2023). A single-item measure of self-rated mental health and psychological distress. In what situations can a single-item measure be useful?. Australas. Psychiatry.

[4] Galambos N.L., Johnson M.D., Krahn H.J. (2022). Self-rated mental health in the transition to adulthood predicts depressive symptoms in midlife. Curr. Psychol..

[5] Wang L., Chen H., Ye B., Gao J., Dai J., Wang F., Fu H. (2019). Mental health and self-rated health status of internal migrant workers and the correlated factors analysis in Shanghai, China: A cross-sectional epidemiological study. Int. Health.

[6] Ahmad F., Jhajj A.K., Stewart D.E., Burghardt M., Bierman A.S. (2014). Single item measures of self-rated mental health: A scoping review. BMC Health Serv. Res..

[7] Jang Y., Park N.S., Kim G., Kwag K.H., Roh S., Chiriboga D.A. (2012). The association between self-rated mental health and symptoms of depression in Korean American older adults. Aging Ment. Health.

[8] Assari S. (2018). Ethnic groups differ in how poor self-rated mental health reflects psychiatric disorders. J. Racial Ethn. Health Disparities.

[9] Kim G., DeCoster J., Chiriboga D.A., Jang Y., Allen R.S., Parmelee P. (2011). Associations between self-rated mental health and psychiatric disorders among older adults: Do racial/ethnic differences exist?. Am. J. Geriatr. Psychiatry.

[10] Han Q., Zheng B., Agostini M., Bélanger J.J., Gützkow B., Kreienkamp J., Reitsema A.M., van Breen J.A., Collaboration P., Leander N.P. (2021). Associations of risk perception of COVID-19 with emotion and mental health during the pandemic. J. Affect. Disord..

[11] Lai D.W., Li J., Bai X. (2021). To be or not to be: Relationship between grandparent status and health and wellbeing. BMC Geriatr..

[12] Jang Y., Huang Y.-C., Yoon H., Lin S. (2016). Correlates of self-rated health and self-rated mental health in older Chinese Americans. Soc. Work Public Health.

[13] Fung H.W., Chien W.T., Ling H.W.H., Ross C.A., Lam S.K.K. (2022). The mediating role of post-traumatic stress disorder symptoms in the relationship between childhood adversities and depressive symptoms in two samples. Child Abus. Negl..

[14] Fung H.W., Wong E.N.M., Lam S.K.K., Chien W.T., Hung S.L., Ross C.A., Cloitre M. (2022). Prevalence and sociocultural correlates of post-traumatic stress disorder (PTSD) and complex PTSD among Chinese community health service users in Hong Kong. Int. J. Soc. Psychiatry.

[15] Sawicki–Luiza P.A.A.A., Atroszko S.B. Validity and reliability of single-item self-report measure of global self-esteem. Proceedings of the CER Comparative European Research.

[16] Robins R.W., Hendin H.M., Trzesniewski K.H. (2001). Measuring global self-esteem: Construct validation of a single-item measure and the Rosenberg Self-Esteem Scale. Personal. Soc. Psychol. Bull..

[17] Kung S., Alarcon R.D., Williams M.D., Poppe K.A., Moore M.J., Frye M.A. (2013). Comparing the Beck Depression Inventory-II (BDI-II) and Patient Health Questionnaire (PHQ-9) depression measures in an integrated mood disorders practice. J. Affect. Disord..

[18] Kroenke K., Spitzer R.L., Williams J. (2001). The PHQ-9: Validity of a brief depression severity measure. J. Gen. Intern. Med..

[19] Manea L., Gilbody S., McMillan D. (2012). Optimal cut-off score for diagnosing depression with the Patient Health Questionnaire (PHQ-9): A meta-analysis. Can. Med. Assoc. J..

[20] Yeung A., Fung F., Yu S.-C., Vorono S., Ly M., Wu S., Fava M. (2008). Validation of the Patient Health Questionnaire-9 for depression screening among Chinese Americans. Compr. Psychiatry.

[21] Leung D.Y., Mak Y.W., Leung S.F., Chiang V.C., Loke A.Y. (2020). Measurement invariances of the PHQ-9 across gender and age groups in Chinese adolescents. Asia-Pac. Psychiatry.

[22] Cloitre M., Hyland P., Prins A., Shevlin M. (2021). The international trauma questionnaire (ITQ) measures reliable and clinically significant treatment-related change in PTSD and complex PTSD. Eur. J. Psychotraumatology.

[23] Ho G.W., Karatzias T., Cloitre M., Chan A.C., Bressington D., Chien W.T., Hyland P., Shevlin M. (2019). Translation and validation of the Chinese ICD-11 International Trauma Questionnaire (ITQ) for the Assessment of Posttraumatic Stress Disorder (PTSD) and Complex PTSD (CPTSD). Eur. J. Psychotraumatology.

[24] Blevins C.A., Weathers F.W., Davis M.T., Witte T.K., Domino J.L. (2015). The Posttraumatic Stress Disorder Checklist for DSM-5 (PCL-5): Development and initial psychometric evaluation. J. Trauma. Stress.

[25] Geier T.J., Hunt J.C., Nelson L.D., Brasel K.J., deRoon-Cassini T.A. (2019). Detecting PTSD in a traumatically injured population: The diagnostic utility of the PTSD Checklist for DSM-5. Depress. Anxiety.

[26] Fung H.W., Chan C., Lee C.Y., Ross C.A. (2019). Using the Post-traumatic Stress Disorder (PTSD) Checklist for DSM-5 to screen for PTSD in the Chinese context: A pilot study in a psychiatric sample. J. Evid.-Based Soc. Work.

[27] Ross C.A., Heber S., Norton G.R., Anderson D., Anderson G., Barchet P. (1989). The Dissociative Disorders Interview Schedule: A structured interview. Dissociation.

[28] Ross C.A., Ellason J.W. (2005). Discriminating among diagnostic categories using the Dissociative Disorders Interview Schedule. Psychol. Rep..

[29] Fung H.W., Ling H.W.H., Ross C.A., Tse J.W.-L., Liu R.K.W. (2020). Dissociative, schneiderian and borderline personality symptoms in a non-clinical sample in Hong Kong: A preliminary report. Eur. J. Trauma Dissociation.

[30] Fung H.W., Choi T.M., Chan C., Ross C.A. (2018). Psychometric properties of the pathological dissociation measures among Chinese research participants—A study using online methods. J. Evid.-Inf. Soc. Work.

[31] Ross C.A., Browning E. (2017). The Self-Report Dissociative Disorders Interview Schedule: A preliminary report. J. Trauma Dissociation.

[32] Fung H.W., Chan C., Lee C.Y., Yau C.K.M., Chung H.M., Ross C.A. (2020). Validity of a web-based measure of borderline personality disorder: A preliminary study. J. Evid.-Based Soc. Work.

[33] Haddock G., McCarron J., Tarrier N., Faragher E. (1999). Scales to measure dimensions of hallucinations and delusions: The psychotic symptom rating scales (PSYRATS). Psychol. Med..

[34] Chien W.T., Lee I.Y.-M., Wang L.-Q. (2017). A Chinese version of the Psychotic Symptom Rating Scales: Psychometric properties in recent-onset and chronic psychosis. Neuropsychiatr. Dis. Treat..

[35] Koo T.K., Li M.Y. (2016). A guideline of selecting and reporting intraclass correlation coefficients for reliability research. J. Chiropr. Med..

[36] Demetriou C., Ozer B., Essau C. (2015). Self-report questionnaires. Encycl. Clin. Psychol..

[37] Bernardi R.A., Nash J. (2023). The importance and efficacy of controlling for social desirability response bias. Ethics Behav..

